# Marijuana use in U.S. teen drivers: a comparison of a road-side survey of reported use and fluid tests for tetrahydrocannabinol (THC)

**DOI:** 10.1186/s40621-019-0204-0

**Published:** 2019-05-29

**Authors:** Joyce C. Pressley, Arushi Arora, Raina Sarmah

**Affiliations:** 1 Columbia University Mailman School of Public Health Departments of Epidemiology and Health Policy and Management, 722 West 168th St., New York, NY 10032 USA; 2 Health Policy and Management, New York, NY USA; 3 Center for Injury Epidemiology and Prevention at Columbia, New York, NY USA

**Keywords:** Marijuana, Tetrahydrocannabinol (THC), Teens, Drivers, Impaired driving, Surveillance, National Roadside Survey (NRS)

## Abstract

**Background:**

Although the growth of state-level legalization of marijuana is aimed at increasing availability for adults and the chronically ill, one fear is that this trend may also increase accessibility in younger populations. The objectives of this study are to evaluate marijuana use in teen driver study participants and to compare their survey self-reported use with oral fluid and blood tests for psychoactive metabolites of tetrahydrocannabinol (THC).

**Methods:**

The National Roadside Survey (NRS) of 2013–2014 was used to examine marijuana use in drivers aged 16–19 years. Of 11,100 drivers surveyed at 300 U.S. locations in 24 states, 718 were 16–19 years, and 666 (92.8%) provided oral fluid and/or blood. We examined weighted and unweighted data, but present unweighted findings. Kappa statistics, Chi square, and multivariable logistic regressions were used to assess agreement, associations and independent predictors of outcomes.

**Results:**

More than one-quarter (203/718) of teen drivers reported either using marijuana in the last year or were THC positive. Overall incidence of a THC positive fluid test was 13.7%. In addition to 175 (27.3%) teen drivers who reported use in the last year, 28 (4.4%) who denied using in the past year, tested positive for THC. Of 45 teen drivers reporting use in the last 24 h, more than two-thirds (71.1%) were THC positive. Disagreement between the oral and blood test for 305 teen drivers who had both tests was 17 (5.6%), with a Kappa of 0.78 (95% CI 0.69–0.88). Of THC-positive drivers, nearly 20% started drinking alcohol by age 14 and more than 70% by age 16. Age, gender- and income-adjusted independent predictors of a positive THC test included survey completion during the school year (OR 3.2, 95% CI 1.6–6.2), survey-reported marijuana use in last year (OR 5.3, 95% CI 3.0–9.2), current smoker (OR 2.1, 95% CI 1.1–3.7), and alcohol consumption before age 16 (OR 2.3, 95% CI 1.1–3.7).

**Conclusions:**

Although specific THC thresholds for safe driving have not been established, taken in the context of teen crash statistics, THC documented impairments and rapidly relaxing marijuana laws, these findings suggest the need for increased vigilance and stepped-up surveillance in teen drivers.

## Background

There is an upward trend in the state-level legalization of marijuana for medicinal and recreational purposes in the context of non-enforcement of federal laws prohibiting marijuana sale and distribution. Some cities, where use for recreational purposes has not yet been “legalized” by their state, have stopped pursuing users for drug law violations (Mueller & Swift, 2018). While laws are aimed at increasing accessibility of marijuana for medicinal and recreational purposes for adults, one fear is that it may also increase availability in younger populations.

Early initiation of marijuana use and its frequent or heavy use has been reported to be associated with higher school dropout rates, lower academic performance, and other negative impacts on school life (Meier et al., 2012; Crean et al., 2011; National Institute on Drug Abuse (NIDA) for Teens, 2018). Other adverse effects of marijuana use particularly on brain function have been reported with regard to thinking clearly, problem solving ability, memory, learning, attention span, coordination, addiction and other serious mental health issues (Meier et al., 2012; Crean et al., 2011; National Institute on Drug Abuse (NIDA) for Teens, 2018; Copeland et al., 2013). Despite its documented negative effects on young brains, more than one-third (38%) of high school students report having tried marijuana (Centers for Disease Control and Prevention (CDC), 2016), with an age gradient ranging from 13.5% for 8th graders to 45.0% for high school seniors (National Institute on Drug Abuse (NIDA) for Teens, 2018).

There are several national surveys that track trends in self-reported use in youth, although the validity of self-reported activities considered illegal has been questioned. Despite challenges in validation of self-reports, tetrahydrocannabinol (THC) is considered an important area for further examination as it has been linked to impaired performance in a number of safe driving functions including reaction time, coordination, attention, judgment and concentration (Bondallaz et al., 2016; Hartman & Huestis, 2013; Hartman et al., 2015).

This study examines factors associated with teen driver marijuana use with data from a roadside data collection design for survey data and fluid tests for psychoactive metabolites of THC. Using survey reports of marijuana use and laboratory THC tests in teen drivers, we examine: 1) factors associated with a positive THC oral fluid or blood test; 2) factors associated with a positive THC test result in drivers who denied recent use; and 3) factors associated with any marijuana use.

## Methods

### Data sources

This study employed the 2013–2014 wave of the National Highway Traffic Safety Administration’s (NHTSA) National Roadside Survey of Alcohol and Drug Use (NRS) (NHTSA, 2008; Kelley-Baker et al., 2015; Kelley-Baker et al., 2017). Of the 11,100 persons aged 16 and over who were eligible to participate in the survey, 8825 completed the survey. Teen drivers aged 16–19 years (*n* = 718) were selected from this larger population.

### Study population

Data were collected anonymously from voluntary participants in 60 sites, each with five different locations, for a total of 300 locations in the contiguous United States (U.S.). Inclusion criteria required drivers to be non-commercial, aged 16 years or older and able to participate in either English or Spanish (Kelley-Baker et al., 2015; Berning et al., 2015).

### Data collection methods

Feasible locations were randomly selected within the geographic area of the local participating jurisdiction. A multistage sampling frame previously employed by the National Automotive Sampling System—General Estimates System was used (NHTSA, 2008). These sites were selected with the aim of being representative of all drivers in the U.S. Data collections were performed in the four main U.S. census regions with cities and counties selected to be representative of three levels of population density. Roadside signs were posted inviting participants to enter a roadside pull out where they could obtain more information on the study and decide whether they wanted to participate voluntarily and anonymously. For study participants who elected to participate at each of the 60 sites, drivers were administered the survey and oral fluid and/or blood were collected during the roadside stop. Additional detail is available in technical reports (NHTSA, 2008; Kelley-Baker et al., 2015). The IRB exempt study analyzed publicly available, deidentified data.

#### Variable classifications

##### Demographic characteristics

**Driver age** was collected and analyzed by year of age (16, 17, 18, and 19) and analyzed categorically as ages 16–17 and 18–19 years. Blood samples were not collected from drivers under age 18 years.

**Gender** was categorized as male or female, with females being the reference group in multivariable statistical analyses.

**Race** was examined as White non-Hispanic, Black/African American non-Hispanic, Hispanic, Asian non-Hispanic, or more than one race/other.

### Geography

Geographic region used U.S. census designations: South, Northeast, Midwest and West.

### Time of year

Surveys for teens aged 16–19 years were collected between June 2013 and March 2014. Month was extracted from the time stamp variable for survey completion and was used to define whether the survey was completed during the months June to August or September to March. Although there are regional differences in the school year, we used the months of June, July and August to approximate summer, and September through March as an approximation of school being in session. There were no data collections in April and May.

### Education

Educational level was available as number of grades completed: less than high school (0–8 grades), some high school (9–11 grades), high school graduate (12 grades) and some college. Most drivers were students in the process of completing their education. Due to small numbers (*n* = 2), we combined 8th grade or less into a category termed less than high school.

### Employment/vocational status

Variables available on employment and student status were collapsed into the categories of: 1) employed, full time; 2) employed, part-time; 3) student, not employed; 4) unemployed and not a student; and 5) other.

### Income

Household income was categorized as less than or equal to $25,000; $25,001 to $50,000; $50,001 to $75,000; $75,001 to $100,000 and $100,001 or greater. Categories were collapsed for multivariable analyses.

### Tobacco use

Tobacco use was self-reported as a dichotomous variable and by recent use in the survey as: 1) never or none in the last year; 2) more than a month; 3) within the past month, but greater than 2 days; 4) within the past 2 days but more than 1 day; and 5) within the last 24 h.

### Alcohol use

Alcohol use was assessed using two variables: age at first use and number of alcohol drinks consumed in an average week. Age at first use of alcohol was categorized as: 1) no use; 2) less than or equal to 15 years; 3) 16–17 years; and 4) 18–19 years. Number of alcohol drinks per week were categorized as: zero to less than one per week, 1–2 drinks, 3–4 drinks, and five or more drinks per week. Due to small numbers, these categories were collapsed for multivariable analyses as shown in the results section tables.

### Survey reported marijuana use

Marijuana use data were collected in these categories: 1) none in the last year or ever; 2) over a month; 3) within the past month, but greater than 2 days; 4) within the past 2 days but more than 1 day; and 5) within the last 24 h. For multivariable analyses, all categories of marijuana use in the last month were collapsed.

### Definition of survey-reported recent use for measuring congruence with THC fluid tests

Recent use was defined dichotomously as having used marijuana within the past month or not having used within the past month. Drivers were categorized as incongruent with their survey reported marijuana use if they denied marijuana use within the past month, but had a positive THC blood or oral fluid test.

### Positive THC test

A positive THC test was defined by NHTSA as a minimum concentration of 2 ng/mL of active THC metabolites in oral fluid or as 1 ng/mL in blood (NHTSA, 2008; Kelley-Baker et al., 2017).

### State medical marijuana law characteristics

The 24 states with surveyed drivers were categorized into three levels based on state-level marijuana law characteristics at the time the survey was administered. Level 1 included 17 states with no medical marijuana laws at the time of the survey: Alabama, Florida, Illinois, Indiana, Iowa, Kansas, Kentucky, Louisiana, Missouri, Nebraska, North Carolina, Ohio, Oklahoma, Pennsylvania, Tennessee, Texas, and Utah. Level 2 states in this study with medical marijuana laws and state-regulated dispensaries included Maryland, Massachusetts, New Jersey, New Mexico, and New York. Level 3 states with medical marijuana laws with additional leniency provisions included California and Michigan. (National Conference of State Legislatures (NCSL), 2018)

### Categorization of THC test results as positive or negative

Drivers aged 16–17 years old were administered only oral fluid tests. Drivers aged 18–19 years were administered both oral fluid and blood tests. Drivers were categorized as positive if they had a positive test from either blood or oral fluid, and negative if both tests were negative, or if the only test performed was negative. Data collection methodology and analysis used ELISA microplate technology to screen samples for THC. Gas-chromatography-mass spectrometry (GC/MS) or liquid-chromatography-mass spectrometry (LC/MS) were used in laboratory THC confirmation.

### Statistical methodology

Descriptive analyses were conducted on unweighted survey and laboratory results. Driver demographic, behavioral characteristics, THC blood and oral fluid tests were analyzed using Chi square or Fisher’s exact for categorical variables. Variable distribution and numerical qualities of all covariates were examined using bivariate unadjusted analyses prior to conducting multivariable modeling. Statistical analyses report odds ratios (OR) with 95% confidence intervals (CI) using unadjusted logistic regression and adjusted multivariable logistic regression. Kappa and interclass correlation were used to assess agreement. Significance of unadjusted bivariate analyses was used to guide our model building for the multivariable logistic regression. (Hosmer Jr et al., 2013) Tests that examined the potential influence of the multi-level data collection sampling frame were not significant. Significance is defined as *p* < 0.05. SAS version 9.4 (SAS Institute Inc., Cary, NC) was used to conduct all analyses. (SAS Institute Inc, 2014)

## Results

### Study population

Of the 718 teen drivers in the NRS, 666 (92.8%) had an oral fluid and/or blood test result for THC. Ninety-one teen drivers (13.7%) tested positive for THC (Table 1).

**Table 1:** Population characteristics for teen driver participants in the National Roadside Survey (NRS), 2013–2014

	THC Test Results (Oral Fluid and/or Blood)^a^			
	Negative Test (all means)	Positive Test (any means)^b^	Total^1^	
	n (%)			Chi Square (*p*-value)
Total (n)	575	91	718	
Demographic Characteristics				
Age (yrs)				0.02
16–17	132 (23.0)	11 (12.1)	154 (21.5)	
18–19	443 (77.0)	80 (87.9)	564 (78.6)	
Gender				0.04
Male	349 (61.0)	65 (72.2)	446 (62.5)	
Female	223 (39.0)	25 (27.8)	268 (37.5)	
Race/Ethnicity				0.28
White, Non-Hispanic	416 (72.4)	69 (75.8)	523 (72.8)	
Black, Non-Hispanic	70 (12.2)	15 (16.5)	91 (12.7)	
Hispanic	64 (11.1)	NR	77 (10.7)	
Asian, Non-Hispanic	NR	NR	19 (2.7)	
More than one/Other	NR	NR	NR	
Region				0.45
South	147 (25.6)	19 (20.9)	182 (25.4)	
Northeast	80 (13.9)	17 (18.7)	108 (15.0)	
Midwest	181 (31.5)	32 (35.2)	223 (31.1)	
West	167 (29.0)	23 (25.3)	205 (28.6)	
Education level				0.025
Less than high school	187 (26.0)	18 (19.8)	174 (26.1)	
High school graduate	176 (30.6)	42 (46.2)	237 (33.0)	
College	235 (40.9)	29 (31.9)	281 (39.1)	
Other/Unknown	NR	NR	11 (1.5)	
Vocational status				0.003
Employed, full time	126 (21.9)	34 (37.3)	175 (24.4)	
Employed, part time	283 (49.2)	38 (41.8)	344 (47.9)	
Not employed, student	89 (15.5)	NR	101 (14.1)	
Unemployed, not student	23 (4.0)	NR	33 (4.6)	
Other	54 (9.4)	NR	65 (8.9)	
Household income				0.004
0 < $25,000	170 (29.6)	41 (45.1)	232 (32.4)	
$25,001 < $50,000	117 (20.4)	20 (22.0)	145 (20.2)	
$50,001 < $75,000	84 (14.6)	12 (13.2)	102 (14.2)	
$75,001 < $100,000	NR	NR	75 (10.5)	
$100,001 or more	77 (13.4)	13 (14.3)	93 (13.0)	
Did not answer	57 (9.9)	NR	70 (9.8)	
Time of year				0.0004
June to August	196 (34.1)	14 (15.4)	210 (31.5)	
September to March	379 (65.9)	77 (84.6)	456 (68.5)	
State Medical Marijuana Law (MML) characteristics				0.03
States without MMLs	379 (65.9)	63 (69.2)	474 (66.0)	
States with MMLs & state-regulated dispensary	75 (13.0)	18 (19.8)	106 (14.8)	
States with MMLs plus leniency provisions	121 (21.0)	NR	138 (19.2)	
Seatbelt^c^				0.40
Lap and shoulder	530 (92.1)	82 (90.1)	662 (92.2)	
Other, belted	40 (7.0)	NR	51 (7.1)	
Not belted	NR	NR	NR	
Passengers (Y,N)				0.009
Absent	293 (51.0)	33 (36.3)	352 (49.0)	
Present	282 (49.0)	58 (63.7)	366 (51.0)	

^a^Oral and blood THC tests were missing for 52 study participants who completed the survey

^b^Oral THC values have a cutoff value of 2 ng/mL & blood THC values have a cutoff value of 1 ng/mL (Kelley-Baker, et.al 2017)

^c^Fisher’s exact test generated *p*-values; *NR* Not reported due to small numbers

### Demographics and positive THC test results

Older teens aged 18 to 19 years were approximately twice as likely to test positive as younger teens aged 16–17 years (15.3% vs. 7.7%, *p* = 0.019). Male drivers tended to be more likely to test positive than females. Both non-Hispanic Black (17.7%) and non-Hispanic White (14.2%) teens tended to have a higher proportion of positive THC tests than did Hispanic (7.3%) or Asian (10.5%) teens. Regional differences ranged from 11.5% in the South to 17.5% in the Northeast, but were not statistically different (*p* = 0.45). Nine drivers 18–19 years old were identified as being positive for THC with their blood test. Disagreement between the oral and blood test for 305 teen drivers who had both tests was 17 (5.6%), with a Kappa of 0.78 (95% CI 0.69–0.88).

### Education and time of year

The highest THC positive educational group was high school graduates who did not report being enrolled in college. Teens were more likely to have a positive THC test during school months (September to March) than in the summer (June to August). Only 15.4% of positive THC tests occurred during the three-month summer timeframe (Table 1).

### Income, employment and positive THC test results

Although numbers were small (*n* = 30), unemployed teens who reported not being a student exhibited the highest proportion of THC positive test results (23.3%) (Table 1). Household income showed an inverse relationship with having a positive THC test, ranging from 10.8% for households with incomes greater than $75,000 to 19.4% for households with incomes of $25,000 or less.

### Tobacco smokers and marijuana test results

**Fig. 1:**
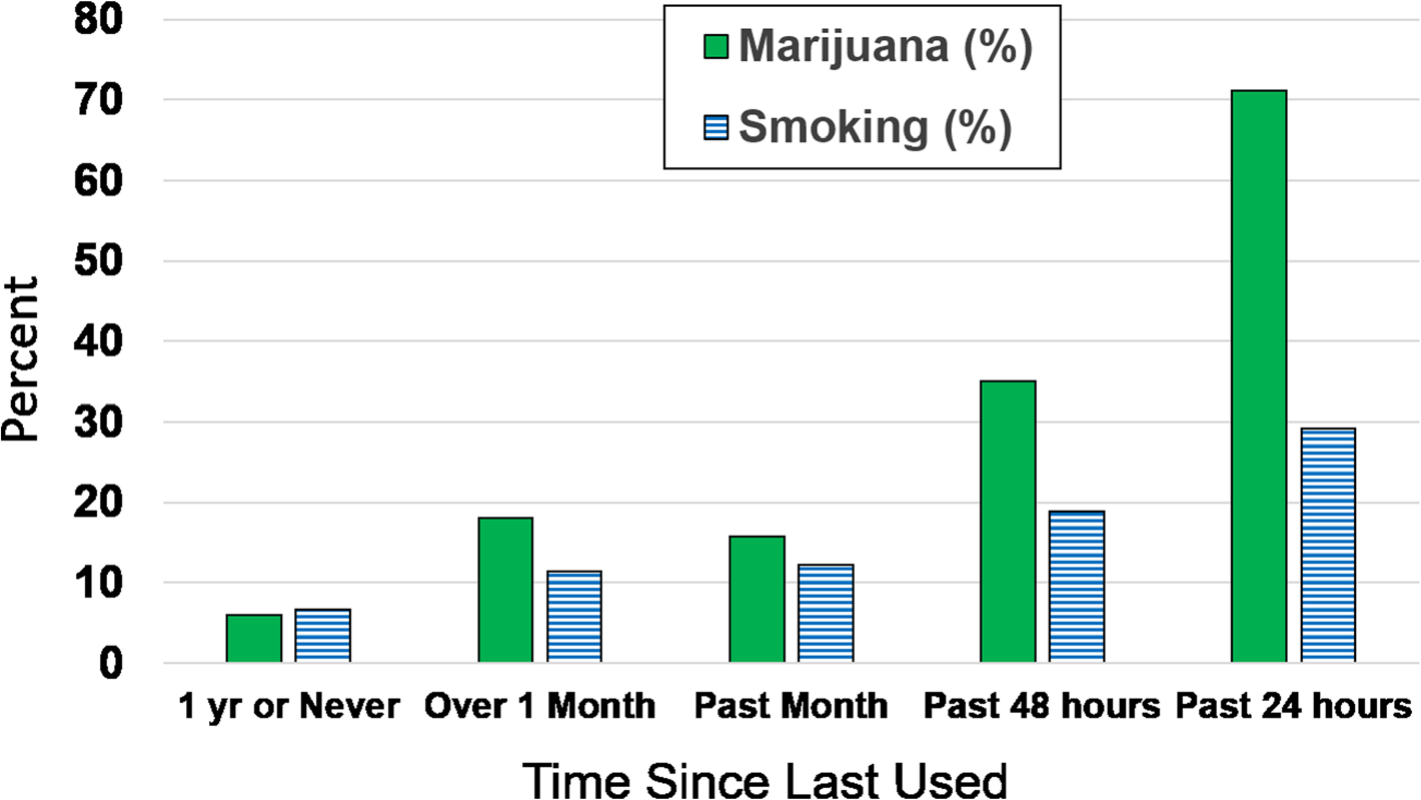
Proportion Testing Positive for THC Within Each Category of Time-Since-Last Used Marijuana and Time-Since-Last-Used Tobacco. Figure Legend 1: The percent positive for THC by time since last used marijuana (shown in solid green) and for time since last used tobacco (shown in blue stripes), National Roadside Survey, 2013–2014

The proportions of positive THC tests were higher in those who reported having smoked tobacco in the last 2 days (18.9%) and in the past 24 h (29.2%). Teen tobacco smoking and having a positive THC test result showed a near linear positive trend (Fig. 1). The lowest proportion of positive THC results (6.6%) was noted in those who reported never or not having smoked tobacco in the last year. For those who reported being smokers, the proportion of positive THC test results was approximately twice that of nonsmokers for both those who reported not having smoked in the last month (11.4%) and those who reported having smoked in the last month (12.2%).

### Age at initiation of alcohol consumption and positive THC test results

**Fig. 2:**
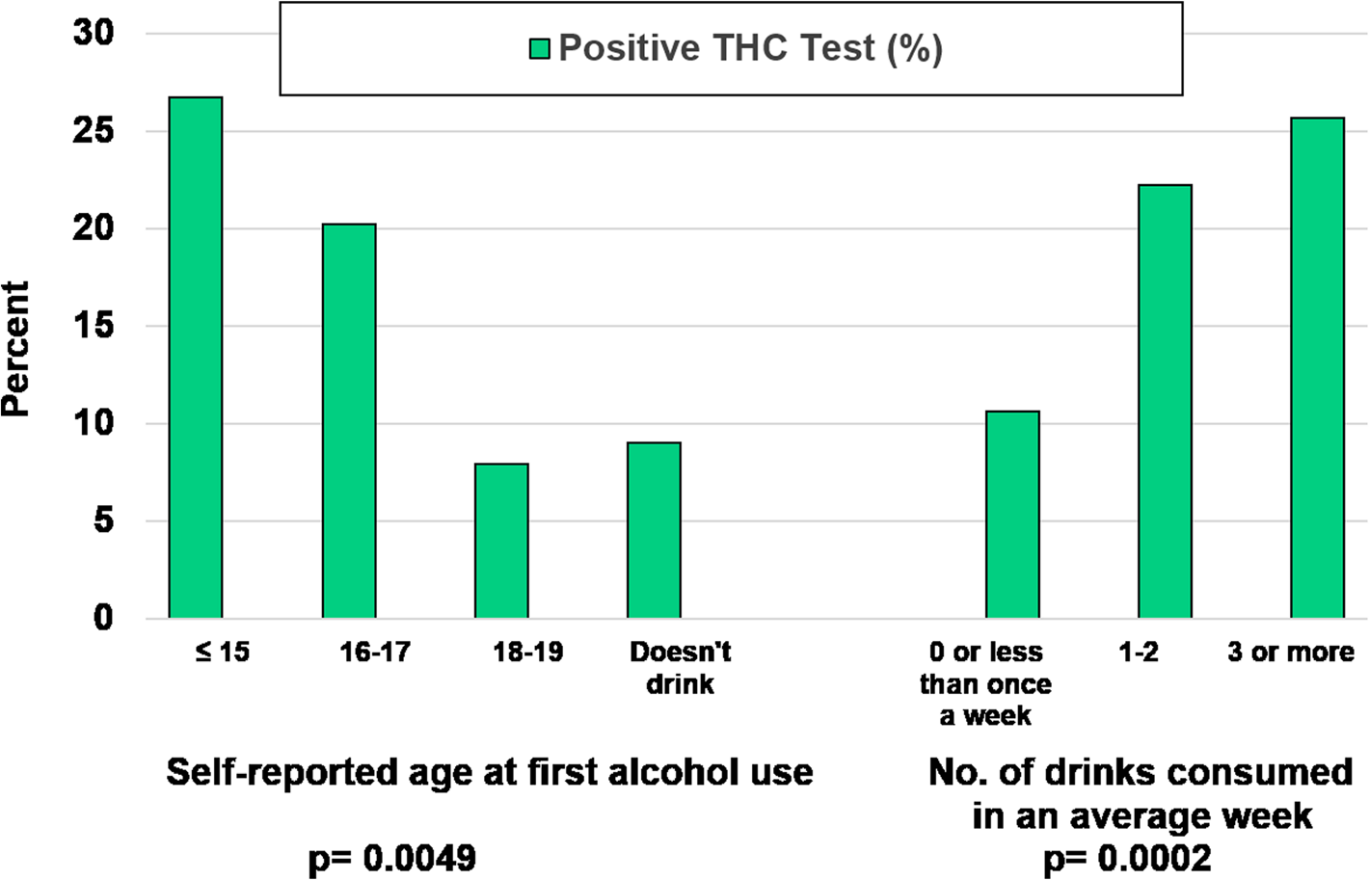
Positive THC Test by Age Began Drinking and Amount Consumed. Figure Legend 2: The percent distribution of a positive THC test (shown in solid green) by age at which the adolescent driver began drinking and number of drinks consumed in an average week, National Roadside Survey, 2013–2014

There was a positive association between initiation of underage drinking at younger ages and having a positive THC test (Fig. 2). The strongest relationship was observed among teen drivers who reported beginning to drink alcohol by age 15 years, where more than one-fourth had a positive THC test result. Teens who initiated drinking alcohol by age 14 years or younger also exhibited a higher than average positive THC test result with more than one quarter (25.7%) testing positive (data not shown). For those who began drinking at age 18 years or later, 8.6% had a positive THC test, which was similar to those who reported not drinking alcohol (9.8%). In bivariate analyses of those with a positive THC test and teens who began drinking before age 16 years, 75% also smoked tobacco.

### Survey reports of recent marijuana use and THC test results

**Fig. 3:**
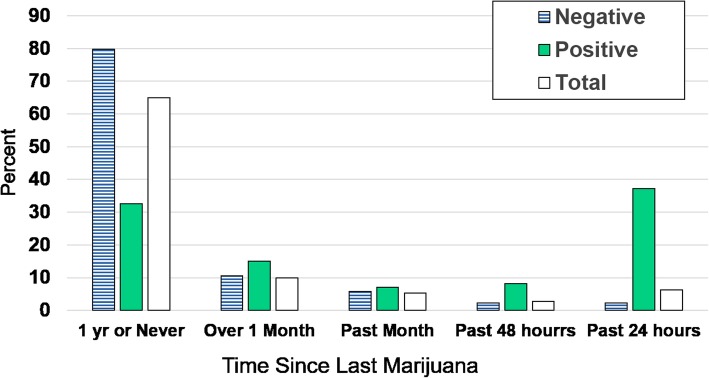
Distribution of THC Positive and Negative Test Results Across Self-Reported Marijuana Use Categories. Figure Legend 3: The percent distribution of positive THC test (shown in solid green), negative THC test (shown in blue stripes) and the Total (shown in white) in adolescent drivers by time since last reported marijuana use, National Roadside Survey, 2013–2014

The distribution of self-reported recent marijuana use by positive and negative fluid tests is shown in Fig. 3. Among teens who tested positive for THC, nearly half denied recent use within the past month (Fig. 3). Within the categories of time since last used marijuana, positive THC test results ranged from 6% (1 year or never) to 71% (in past 24 h). The proportion of THC positive drivers increased steadily among those who reported not having used in the last month (18.1%), used in the past month (15.8%), used in the past 2 days (35.0%) and used in the last 24 h (71.1%).

### Driving characteristics and presence of passengers

Seat belt use was high and similar across survey reported marijuana users and THC test positive drivers. Among teens with a positive THC test, nearly two-thirds were transporting passengers compared to approximately half of THC negative teen drivers. Among all teens with a passenger present in the vehicle, 17.1% had a positive THC test result compared to those traveling alone (10.1%) (*p* = 0.009).

### Independent predictors of teen drivers having a positive THC test

Several factors were associated with increased odds of having a positive oral fluid or blood THC test (Table 2). Model 1 was self-reported recent marijuana use, adjusted for demographics (age, gender, and household income). Three of four timeframes indicating use within the last year were significantly predictive of having a positive THC blood or oral fluid test, with the two most recent reported use categories being associated with higher odds of having a positive test. In an age-, gender- and income-adjusted model (Model 2), a dichotomous variable for having tried marijuana in the last year (Yes/No), any tobacco use in the last year (Yes/No), and began drinking alcohol at age 15 years or younger were all independently predictive of having a positive fluid test for THC. When the time of survey/testing was added to model 2, (shown as Model 3), having been surveyed during school months September to March was associated with triple the odds of having a positive oral or blood test compared to survey respondents from the summer months of June to August.

**Table 2:** Predictors of positive THC test among adolescent drivers: Unadjusted and adjusted odds ratios^a^

	Unadjusted OR (95% CI)	Multivariable Adjusted OR^b^ (95% CI)		
		Model 1^c^	Model 2^d^	Model 3^e^
Demographic Characteristics				
Age (yrs)				
16–17	Ref	Ref	Ref	Ref
18–19	2.17 (1.12, 4.19)	1.34 (0.61, 2.97)	1.19 (0.57, 2.52)	1.21 (0.57, 2.57)
Gender				
Female	Ref	Ref	Ref	Ref
Male	1.66 (1.02, 2.71)	1.81 (0.99, 3.32)	1.64 (0.93, 2.89)	1.58 (0.88, 2.80)
Household income				
0 < $25,000	Ref	Ref	Ref	Ref
$25,001 < $50,000	0.71 (0.40, 1.27)	0.90 (0.45, 1.82)	0.90 (0.47, 1.74)	0.85 (0.44, 1.67)
$51,001 < $75,000	0.59 (0.30, 1.187)	0.79 (0.35, 1.81)	0.62 (0.28, 1.38)	0.55 (0.24, 1.25)
$75,001 and more	0.45 (0.24, 0.84)	0.42 (0.20, 0.92)	0.47 (0.24, 0.93)	0.46 (0.23, 0.92)
Substance use				
Self-reported marijuana use				
Beyond a year/Never	Ref	Ref		
Over a month	3.45 (1.69, 7.02)	3.31 (1.48, 6.64)		
Past month	2.93 (1.13, 7.60)	2.38 (0.84, 6.74)		
Past 2 days	8.42 (3.11, 22.78)	8.28 (2.88, 23.81)		
Past 24 h	38.51 (18.20, 81.46)	40.21 (18.02, 89.74)		
Current Marijuana Use				
Beyond a year/Never	Ref		Ref	Ref
Used in last year			5.60 (3.23, 9.73)	5.28 (3.01, 9.24)
Tobacco use				
Beyond a year/Never	Ref		Ref	Ref
Current user	4.22 (2.56, 6.96)		1.81 (1.02, 3.23)	2.05 (1.13, 3.70)
Self-reported age at first alcohol use (yrs)				
Does not drink/After 15	Ref		Ref	Ref
≤ 15	3.02 (1.70 5.38)		2.17 (1.12, 4.19)	2.27 (1.13, 3.70)
Time of year				
June–August	Ref			Ref
September–March	2.84 (1.57, 5.16)			3.18 (1.60, 6.21)

^a^THC levels in oral fluid test or blood test, National Roadside Survey (NRS), 2013–2014

^b^Race/ethnicity, census region, presence or absence of a medical marijuana law, and educational levels were tested in all models, but were dropped from all models due to lack of significance

^c^Model 1 included age, gender, household income and time since self-reported marijuana use

^d^Model 2 included age, gender, household income, marijuana use in last year (Y/N), tobacco use in last 24 h, and began drinking alcohol by age 16 years

^e^Model 3 added time of year of survey/fluid testing to variables in model 2 (age, gender, household income, marijuana use in last year (Y/N), tobacco use in last 24 h, and began drinking alcohol by age 16 years)

### Independent predictors of teen drivers who denied use, but had a positive THC test

Independent predictors of having a positive THC test were assessed in a subset of teen drivers who reported not having used marijuana recently (1 month or longer), but who had a positive THC test result (Table 3). Significant predictors for denial of marijuana use with a positive THC test included completing a survey conducted during school months, tobacco use in the last 24 h, passengers present  and being surveyed in a state having either no state medical marijuana law or one with more stringent controls on their state medical marijuana law in comparison to states with state medical marijuana laws that had extra leniency legal provisions. There were no differences in the consistency between last reported marijuana use and THC test results for demographic variables, education, income, employment, region of the country or alcohol consumption.

**Table 3:** Predictors of a positive THC test in survey reported non-users: Unadjusted and adjusted odds ratios^a^

	Unadjusted OR (95% CI)	Multivariable Adjusted OR (95% CI)
Demographic Characteristics		
Age (yrs)		
16–17	Ref	
18–19	1.84 (0.75, 4.47)	
Gender		
Female	Ref	
Male	1.86 (0.89, 3.90)	
Race/Ethnicity		
White, Non-Hispanic	Ref	
Black, Non-Hispanic	1.58 (0.69, 3.59)	
Hispanic	0.41 (0.10, 1.78)	
Region		
South	Ref	
Northeast	2.65 (0.88, 7.96)	
Midwest	2.40 (0.93, 6.20)	
West	1.09 (0.37, 3.23)	
Education level		
Less than high school	Ref	
High school graduate	1.38 (0.62, 3.04)	
College	0.81 (0.35, 1.89)	
Other	1.60 (0.18, 14.0)	
Vocational status		
Employed, full time	2.04 (0.98, 4.28)	
Employed, part time	Ref	
Not employed, student	1.0 (0.36, 2.81)	
Unemployed, not student	2.27 (0.61, 8.45)	
Other	0.63 (0.14, 2.83)	
Household income		
0 < $25,000	Ref	
$25,001 < $50,000	0.78 (0.33, 1.82)	
$51,001 < $75,000	0.82 (0.33, 2.06)	
$75,001 or more	0.55 (0.23, 1.32)	
Time of year		
June–August	Ref	Ref
September–March	2.50 (1.09, 5.76)	3.04 (1.28, 7.24)
Substance use		
Tobacco use		
Beyond a year/Never	Ref	Ref
Within a month/Year	1.07 (0.39, 2.94)	1.11 (0.40, 3.10)
Past 24 h	2.65 (1.32, 5.31)	3.49 (1.67, 7.27)
Number of alcoholic drinks consumed in an average week		
0 or less than once a week	Ref	
1–2 times	1.16 (0.39, 3.41)	
3–4 times	3.55 (0.71, 17.74)	
Self-reported age at first alcohol use (yrs)		
No use	Ref	
≤ 15	2.39 (0.97, 5.91)	
16–17	1.40 (0.58, 3.38)	
18–19	0.86 (0.25, 2.96)	
State Medical Marijuana Law (MML) characteristics		
States without MMLs	3.44 (1.03, 11.49)	3.67 (1.08, 12.46)
States with MMLs & state-regulated dispensaries	4.17 (1.04, 16.71)	5.0 (1.20, 20.76)
States with MMLs & additional leniency provisions	Ref	Ref
Passengers (Y, N)		
Absent	Ref	Ref
Present	2.22 (1.12, 4.38)	2.42 (1.20, 4.89)

^a^National Roadside Survey (NRS), 2013–2014

### Independent predictors of teen marijuana use

Total marijuana use was examined in those who had either a positive THC test result or who reported being a user (*n* = 203) compared to nonusers (never or not in the last year) (*n* = 438). Independent predictors of being a teen driver who had a history of having tried marijuana included: alcohol consumption of 1–2 times per week (OR 2.79, 95% CI 1.63–4.76), 3 or more times per week (OR 2.78, 95% CI 1.30–5.92), tobacco use (more than 1 day and less than 1 month) (OR 4.39, 95% CI 2.74–7.03) or tobacco use (within the past 24 h) (OR 4.59, 95% CI 2.97–7.08).

## Discussion

In this study, nearly one-third of teen drivers reported having tried marijuana and nearly 14% of teen drivers had a positive THC oral fluid or blood test at the time of the roadside survey. Depending on age, other studies report lifetime use by teens to be as high as 38%–45%, with 5.5%–22.9% reporting use in the past month and as many as 5.9% reporting daily use. In this study, we were not able to assess chronic daily use, but more than 7% reported having used in the last 24 h. Our findings are consistent other reports of teen marijuana use (National Institute on Drug Abuse (NIDA) for Teens, 2018; Centers for Disease Control and Prevention (CDC), 2016; Miech et al., 2017; Center for Behavioral Health Statistics and Quality (CBHSQ), 2015).

Teens who used marijuana were multi-substance users who were also more likely to smoke and drink compared to teen drivers who tested negative for THC. Early use of alcohol was associated in bivariate analyses with teens being THC positive, particularly for those who report beginning to drink before age 16 years. There are reports of early onset of use being associated with increased risks of addiction, with rates of marijuana addiction being higher in users who begin using before age 18 years (National Institute on Drug Abuse (NIDA) for Teens, 2018; Centers for Disease Control and Prevention (CDC), 2016). These findings indicate a clustering of substance use with both alcohol and tobacco consumption. Drivers who were positive for THC at their roadside test were twice as likely to be current tobacco smokers than drivers who were negative for THC. However, we did not have information on whether mode of use was inhalation or ingestion.

Although having a positive THC test cannot be equated with being impaired by marijuana, many of the driving skills that are just developing in teens and that have been shown to impact crash risk are also negatively impacted by THC (Bondallaz et al., 2016; Hartman & Huestis, 2013). Marijuana has been shown to impair judgment, coordination, reaction times and other mental and related physical functions needed for safe driving (Hartman & Huestis, 2013; Hartman et al., 2015; Lenné et al., 2010).

In addition, there are several conflicting studies on whether marijuana use is associated with an increased crash risk. One issue with evaluating the association between crash risk and THC is that, unlike alcohol, standards for impairment are not yet available. The presence of THC may linger in body fluids for days despite the fact that the psychoactive influence of THC may dissipate within hours of use. Thus, even though nearly 14% of teen drivers in this study were THC positive, we have no measures that can indicate or confirm impairment that would affect driving. It is possible that the discrepancies in findings of crash risk may be dependent on whether the study included only recent users who still had psychoactive THC on board, such as in driving simulator studies, or a greater mix of users who were positive, but had not consumed marijuana recently. NHTSA commissioned a prospective study that showed an overall 25% higher risk of crash, but once they controlled for the higher crash risk associated with younger age and male drivers, THC crash risk was no longer statistically significant. Other studies of drivers in very serious or fatal crashes have found crash effects that were both larger and smaller than those noted in the NHTSA study (Compton, 2017).

We observed some regional differences in unadjusted analyses of teen marijuana use. Although we did not find evidence that medical marijuana laws increased teen access to marijuana, we did find evidence that suggests an association between features of the laws and congruence between a teen driver’s survey report of marijuana use and positive THC test results. Further study is needed to examine the observation that state medical marijuana laws with additional leniency legal provisions appear to be associated with increased congruence, possibly “truthfulness” of reporting on their use of marijuana. While this could have implications for surveys such as those collected through the Youth Risk Behavior Surveillance System (YRBSS) and other surveys that assess school-age marijuana use in the absence of blood or saliva confirmatory tests, we were unable to assess the possibility that a positive THC test resulted from consumption of tainted food or from second-hand marijuana smoke (Cone et al., 2015; Compton & Berning, 2015).

Several obstacles have been reported with regard to establishing standards that have implications for interpreting the positive THC levels in the teen drivers in this study. Low THC levels can be associated with high impairment and vice versa, and low levels can be found in chronic users who have no impairment (Compton, 2017). In a report to congress, NHTSA noted that THC is fat-soluble, has different elimination properties, and behaves very differently in our currently available drug tests than water-soluble alcohol. THC can be stored in fat tissues and released back into the blood up to 30 days post ingestion (Compton, 2017). Marijuana’s psychoactive effects on driving are thought to last for hours, rather than days or weeks, although the length of psychoactive properties on an individual are reported to be affected by whether it is smoked, drank, or eaten (Compton, 2017).

This study has limitations. Although there are more than 100 marijuana metabolites detectable in blood, there are reports that only two of these are psychoactive when distributed to various areas of the body including the brain (Compton, 2017). Inclusion of the fluid verification for presence of THC is a strength of this study that is not present in self-report only studies. However, there are no well-established safe driving thresholds for either delta 9 tetrahydrocannabinol or 11-OH-THC, which are the two psychoactive compounds used to define a positive THC test (Kelley-Baker et al., 2015; Compton, 2017). This study sample is composed of road-side volunteer drivers who were compensated for participation. It is possible that misclassification of a small number of 16–17 year olds could have resulted from younger drivers aged 16 and 17 years receiving only oral fluid tests, while 18 and 19 year olds had both oral fluid and blood testing. A small group (*n* = 9) of THC positive 18 and 19 year old drivers was identified only through blood testing. In addition, while the oral fluid THC test is being used widely in other countries, at the time of this study, it was not yet in widespread use in the U.S. (Lee & Huestis, 2014; MacDonald et al., 2014).

Unlike alcohol that is cleared from the human body more readily and where there are well established limits for impairment, THC does not have gold-standard established guidelines for impairment and THC remains detectable in body fluids long after its impairment has ceased (Compton, 2017; Van der Linden et al., 2013). The detection of THC has been reported to linger in chronic users for longer than short-term or occasional users. Another factor reported to complicate these issues is that the THC can be laced in food as well as inhaled. The mode of intake has been reported potentially to differ with regard to the likelihood of a positive test between oral fluid and blood. Given that marijuana can be laced in food, it is possible for someone to be given cannabis without his or her knowledge. Furthermore, there is little information regarding the relation between THC test results and second-hand smoke. Lastly, this study does not attempt to draw conclusions regarding the entire U.S. teen driving population as we did not weight the data to be representative of the national population of teens.

## Conclusions

This study found a significant proportion of teen drivers to be positive for THC. It also found that nearly half of positive THC tests occurred in teens who denied recent marijuana use, and nearly one-third of positives denied use in the last year. This finding may have implications for the interpretation of surveys of teen marijuana use where fluid confirmation is not available. Teen drivers have higher than average crash statistics as many are still developing their driving skills and have mental judgment capacity that is still maturing. Thus, while no specific THC thresholds have been established for operating a motor vehicle, taken in the wider context of teen crash statistics, documented impairments associated with THC and the rapid state-level shifts in marijuana laws, these findings suggest the need for increased vigilance and stepped-up surveillance of THC in teen drivers.

## Abbreviations

11-OH-THC: 11-hydroxy-tetrahydrocannabinol
ELISA: Enzyme-linked immunosorbent **assay**
GC/MS: Gas-chromatography-mass spectrometry
LC/MS: Liquid-chromatography-mass spectrometry
MML: State medical marijuana law
NHTSA: National Highway Traffic Safety Administration
NRS: National Roadside Survey of Alcohol and Drug Use
THC: Tetrahydrocannabinol
